# Incidence and Mortality Trends and Risk Prediction Nomogram for Extranodal Diffuse Large B-Cell Lymphoma: An Analysis of the Surveillance, Epidemiology, and End Results Database

**DOI:** 10.3389/fonc.2019.01198

**Published:** 2019-11-12

**Authors:** Xuejiao Yin, Aoshuang Xu, Fengjuan Fan, Zhenli Huang, Qianwen Cheng, Lu Zhang, Chunyan Sun, Yu Hu

**Affiliations:** ^1^Institute of Hematology, Tongji Medical College, Union Hospital, Huazhong University of Science and Technology, Wuhan, China; ^2^Collaborative Innovation Center of Hematology, Huazhong University of Science and Technology, Wuhan, China

**Keywords:** extranodal DLBCL, incidence, incidence-based mortality, nomograms, prognosis

## Abstract

**Background:** DLBCL is the most commonly occurring type of non-Hodgkin's lymphoma, which may be found at various extranodal sites. But little is known about the particular trends of extranodal DLBCL.

**Methods:** A total of 15,882 extranodal DLBCL patients were included in incidence analysis from the Surveillance, Epidemiology, and End Results (SEER) database (1973–2015). The joinpoint regression software was used to calculate the annual percent change (APC) in rates. Nomograms were established by R software to predict overall survival (OS).

**Results:** The extranodal DLBCL incidence continued to rise at a rate of 1.6% (95% CI, 0.4–2.8, *p* < 0.001) per year over the study period, until it declined around 2003. The incidence-based mortality trend of extranodal DLBCL had a similar pattern, with a decrease happening around 1993. Five-year survival rates improved dramatically from the 1970s to 2010s (44.15 vs. 63.7%), and the most obvious increase occurred in DLBCL patients with primary site in the head/neck. The C-index showed a value for OS of 0.708, which validated the nomograms performed well and were able to forecast the prognosis of patients with extranodal DLBCL. The calibration curves showed satisfactory consistency between true values and predicted values for 1-, 5-, and 10-year overall survival, respectively.

**Conclusions:** The incidence and incidence-based mortality of extranodal DLBCL had been increasing for decades, followed by a promising downward trend in recent years. These findings may help scientists identify disease-related risk factors and better manage the disease. The prediction signature cloud identifies high-risk patients who should receive effective therapies to prevent the fatal nature of this disease, and low-risk patients to reduce over-treatment.

## Introduction

Non-Hodgkin lymphoma (NHL) is the tenth most common cancer in the world in 2018 (1), while the most common NHL subtype is diffuse large B-cell lymphomas (DLBCL), comprising approximately 30% of NHL (2). Lymphoma can arise in any tissue, and approximately one third of patients present with extranodal sites (3). In the United States, NHL incidence has increased about 80% from 1975 to 2003, and extranodal NHL accounts for the most of new cases (4). Etiological exposure, as well as the pattern of incidence and mortality may vary according to anatomical sites. Existing data describing the incidence and mortality trends of DLBCL failed to specially depict extranodal DLBCL, consider the effects of tumor property and demographic character of patients, or make systemic comparison of incidence and mortality trend based on these characteristics (5).

In recent years, the most of DLBCL patients' survival rate have been dramatically increased since R-CHOP (rituximab with cyclophosphamide, doxorubicin, vincristine, and prednisone) was introduced as a first-line therapy (6). However, treatment-related side effects and long-term complications have also been increased, and a significant proportion of patients do not respond to initial treatment or relapse ultimately. At present, the most routine prognostic system in DLBCL patients is the International Prognostic Index (IPI). The number of extranodal sites is one of the evaluation standard of the IPI score. However, Lu CS et al. had indicated that the particular extranodal sites show a better predictive value than the number of extranodal sites involved (7). Besides, there is increasing evidence that primary extranodal sites reflect distinct clinical features and prognostic implications, and require specific therapy (8, 9). Therefore, a new risk stratification signature that includes extranodal sites involved of origin is needed to guide the treatment of extranodal DLBCL. As a graphical expression of a mathematical model, nomographs could combine different information of several features to forecast a specific outcome in clinical practice. By integrating various significant factors, a nomograph could estimate the feasibility of an event for each patient, such as the possibility of death or recurrence. Thus, the nomograph has evolved into an important instrument for forecasting the clinical outcomes of various type of cancer and could provide optimal therapy schemes for physicians.

The present study aimed to explore the incidence and mortality trends of extranodal DLBCL by primary site involved and patient demographic characteristics from the Surveillance, Epidemiology, and End Results (SEER) cancer registry during 1973–2015. The results may help scientists identify disease-related risk factors and better manage the disease. In addition, we have developed a new prediction model specifically for patients with extranodal DLBCL in the rituximab era that may provide an accurate risk stratification of individual and guide the treatment.

## Materials and Methods

### Data Sources

#### Patients to Estimate Incidence and IBM:Seer 9

The incidence cases come from the registries of the SEER-9 cancer incidence file of the US National Cancer Institute from 1973 to 2015. For the SEER database has only been recorded on the Ann Arbor Stage since 1983, we limited the analysis of the impact of the Ann Arbor Stage on the incidence from 1983.

The incidence-based mortality (IBM) cases were different from traditional mortality cases, which linked mortality records to incident cancer cases. The IBM cases were only included from 1988 to 2015 to ensure maximum majority of the deaths occurring after 1973 and IBM rates was not underestimated in the first few years. This interval was chosen, because the mean survival time for patients with DLBCL is 10 years.

#### Patients to Estimate Survival and Construction the Nomogram:SEER 18

The patients diagnosed from 1973 to 2015 were enrolled from the SEER 18 registries with 28% of the US population in the survival analysis.

The patients diagnosed from 2002 to 2015 were selected out from the SEER 18 registries for construction of nomogram. The patients diagnosed before 2002 were excluded, because we aimed to build a prediction model for the rituximab era and 79% of DLBCL patients have received rituximab as first-line treatment from 2002 (10).

### Study Population Selection

Eligible patients were diagnosed with lymphoma with the International Classification of Diseases for Oncology, third edition, ICD-O-3 histology code 9680 (diffuse large B-cell lymphoma, not otherwise specified). These patients were excluded: (1) DLBCL wasn't primary malignancy; (2) the patients were not active follow-up; (3) the primary site of the lymphoma was central nervous system, mediastinum or unknown. Patients with primary CNS DLBCL and primary mediastinal DLBCL were excluded, because they have unique clinical features, prognosis, and the treatment that differ from other sites. The sparse number of patients whose race was unknown were excluded for further evaluation in the incidence and Incidence-Based Mortality (*n* = 66 and *n* = 7, respectively) analysis. In addition, those who survived for less than a month were excluded in survival analysis and construction the nomogram, because their survival time were recorded as 0 in SEER database. We excluded the patients whose Ann Arbor Stage and race was unknown when constructing the nomogram.

### Definition of Variables

Patients' age at diagnosis, sex, race, clinical stage, primary site of involvement, survival time, and cause of death were collected. Patients were divided into 4 groups based on age at diagnosis: (1) children: ≤14 years; (2) AYAs: 15–39 years; (3) adults: 40–64 years; (4) the elderly: ≥65 years, according to the National Cancer Institute Progress Review Group on AYA Oncology and the National Comprehensive Cancer Network guidelines. Autologous stem cell transplantation (ASCT) is an effective method to treat recurrent patients, but ASCT was most commonly offered to patients <65 years. Survival rates change dramatically with age for those older than 65 due to geriatric syndromes and comorbid diseases. Therefore, the elderly were further divided into groups every 5 years in the process of survival analysis and modeling. The stage was divided into early (Ann Arbor Stage I/II) or advanced (III/IV) stage. Based on the ICD-O-3 topography code published by SEER, primary sites were classified into 10 sites: Skin/soft tissue (C44-44.9, C49-49.9); Gastrointestinal tract (C15-21.8, C26-26.9); Head/Neck (C00-14.8, C30-32.9, C73.9, C76); Genitourinary tract (C51-68.9); Skeletal tissue (C40-41.9, C76.3-76.5); Respiratory system (C33.9-34.9, C39-39.9); Hematologic system (C42-42.4); Liver/pancreas (C22-25.9); Breast tissue (C50-50.9); and Other (C48-48.8, C69-69.9, C74-75.9, C76.1-76.2).

### Statistical Analysis

SEER^*^Stat version 8.3.2 was used to calculate incidence and IBM rates. All rates were age adjusted to the 2000 US standard population and expressed per 100,000 person-years. Joinpoint regression analysis program, version 4.5.0.1, was used to analyze the incidence and mortality trends of extranodal DLBCL, and annual percentage change (APC) and average annual percentage change (AAPC) were used to assess rate changes. We use the rms package in R Bioconductor to calculate 1-year, 5- and 10-year overall survival (OS). In order to determine how different variable levels were associated relatively, as well as individually, with survival, Univariable and multivariable Cox proportional hazards regression models were used to calculate hazard ratios (HR) and 95% confidence intervals (CI). SPSS used for survival analyses. *P* < 0.5 was a statistically significant standard.

### Construction and Validation of the Nomogram

The steps to construct the nomogram are as follows. Firstly, the primary outcome was set to be OS. Secondly, variables (e.g., age at diagnosis, sex, race, clinical stage, and primary site of involvement) which may determine the outcome were selected on basis of priori clinical hypotheses. Thirdly, the survival related factors were determined via Cox proportional hazards regression model. Finally, a prognostic nomogram for OS was built through R Bioconductor based on multivariate analysis above.

As for the application of the nomogram, firstly, the point for each trait of the patient was allocated via a vertical line from the corresponding variable to the point scale. Then, all the points were summed up and a vertical line was drawn from the total point scale to obtain different probability of 1-, 5-, and 10-year OS.

Concordance index (C-index) and calibration curve are used to assess the performance of the model. C-index >0.5 is considered statistically significant, and a larger value indicates a stronger predictive ability of the model. The closer the predicted value is to the diagonal line on the calibration plot, the stronger the model prediction ability. The model experienced 1,000 bootstrap reiterations.

## Results

### Patient Characteristics in Incidence and Mortality Analysis

A total of 15,882 patients with extranodal DLBCL as the first malignancy diagnosed were included in the incidence analysis from SEER database from 1973 to 2015. Patient characteristics are outlined in Table 1. Among them, man (8,620 [54.275]), early stage (9,841 [61.963]), the elderly (8,776 [55.258]), and white groups (13,270 [83.554]) comprised the largest proportion in patients included. The most common primary sites were gastrointestinal tract (5,430 [34.190]), head/neck (3,602 [22.680]), and skin and soft tissue (1,717 [10.811]). Of these patients, 8,776 died of extranodal DLBCL from 1988 to 2015 and were included in the incidence-based mortality analysis. Most of the deaths occurred in man (4,745 [54.068]), early stage (5,377 [61.269]), the elderly (6,795 [77.427]), and white group (7,437 [84.742]). Trends of incidence and incidence-based mortality by tumor characteristics were described in Tables 2, 3, respectively.

**Table 1:** Extranodal diffuse large B-cell lymphoma incidence (1973–2015) and incidence-based mortality (1988–2015): the SEER-9 registry database.

**Characteristic**	**Incidence**		**Incidence-based mortality**	
	**Cases, No. (%)**	**Rate (95% CI)**	**Cases, No. (%)**	**Rate (95% CI)**
**Overall**	15,882	1.579 (1.555–1.604)	8,776	1.205 (1.179–1.230)
**Age, y**				
≤14	93 (0.586)	0.041 (0.033–0.50)	6 (0.068)	0.004 (0.001–0.008)
15–39	1,409 (8.872)	0.359 (0.340–0.378)	352 (4.011)	0.129 (0.116–0.143)
40–64	5,604 (35.285)	1.742 (1.696–1.788)	1623 (18.494)	0.682 (0.649–0.716)
65–85+	8,776 (55.258)	7.230 (7.079–7.384)	6795 (77.427)	7.522 (7.344–7.703)
**Sex**				
Male	8,620 (54.275)	1.934 (1.893–1.976)	4745 (54.068)	1.589 (1.543–1.635)
Female	7,262 (45.725)	1.289 (1.260–1.320)	4031 (45.932)	0.923 (0.895–0.952)
**Race^#^**				
White	13,270 (83.554)	1.596 (1.568–1.623)	7437 (84.742)	1.233 (1.205–1.261)
Black	967 (6.089)	1.041 (0.974–1.111)	491 (5.595)	0.772 (0.7–2–0.845)
Other	1,579 (9.942)	1.797 (1.708–1.889)	841 (9.583)	1.235 (1.152–1.323)
Ann Arbor Stage^*^				
I/II	9,841 (61.963)	1.175 (1.152–1.198)	5,377 (61.269)	0.738 (0.718–0.758)
III/IV	3,962 (24.946)	0.474 (0.459–0.489)	2,553 (29.091)	0.350 (0.337–0.364)
Unknown	749 (4.716)	0.089 (0.083–0.096)	846 (9.640)	0.116 (0.109–0.124)
**Site**				
Head/Neck	3,602 (22.680)	0.359 (0.347–0.371)	1,842 (20.989)	0.253 (0.241–0.264)
Skin and soft tissue	1,717 (10.811)	0.171 (0.163–0.179)	944 (10.757)	0.130 (0.122–0.138)
Gastrointestinal tract	5,430 (34.190)	0.541 (0.527–0.556)	3,235 (36.862)	0.444 (0.429–0.460)
Genitourinary tract	1,175 (7.398)	0.116 (0.110–0.123)	629 (7.167)	0.086 (0.080–0.094)
Skeletal tissue	963 (6.063)	0.094 (0.088–0.100)	387 (4.410)	0.053 (0.048–0.059)
Respiratory system	681 (4.288)	0.068 (0.063–0.073)	438 (4.991)	0.060 (0.054–0.066)
Hematologic system	733 (4.615)	0.072 (0.067–0.078)	378 (4.307)	0.051 (0.046–0.057)
Liver/pancreas	577 (3.633)	0.058 (0.053–0.063)	375 (4.273)	0.052 (0.047–0.057)
Breast tissue	423 (2.663)	0.042 (0.038–0.047)	223 (2.541)	0.031 (0.027–0.035)
Other	581 (3.658)	0.058 (0.053–0.063)	325 (3.703)	0.044 (0.040–0.049)

# *The sparse number of patients whose race was unknown were excluded for further evaluation in the incidence and Incidence-Based Mortality (n = 66 and n = 7, respectively) analysis. Therefore, the percentages of race in the incidence and Incidence-Based Mortality analysis do not add up to 100%*.

* *The incidence cases come from the registries of the SEER-9 cancer incidence file of the US National Cancer Institute from 1973 to 2015, while the Ann Arbor Stage case has only been recorded since 1983. Therefore, the percentages of Ann Arbor stages in the incidence analysis do not add up to 100%*.

**Table 2:** Trends in extranodal diffuse large B-cell lymphoma incidence rates (1973–2015): the SEER-9 registry database.

		**Trend**											
	**Overall (1973–2015)**	**1**		**2**		**3**		**4**		**5**		**6**	
	**APC (95% CI)**	**Year**	**APC (95% CI)**	**Year**	**APC (95% CI)**	**Year**	**APC (95% CI)**	**Year**	**APC (95% CI)**	**Year**	**APC (95% CI)**	**Year**	**APC (95% CI)**
**Overall**	1.6 (0.4 to 2.8)	1973–1979	−2.2 (−5.9 to 1.6)	1979–1985	10.3 (6.0–14.8)	1985–1993	5.2 (3.3 to 7.1)	1993–1996	−4.3 (−14.6 to 7.2)	1996–2003	3.3 (1.4 to 5.2)	2003–2015	−2.2 (−2.8 to −1.6)
**Age, y**													
≤14	1.5 (0.2 to 2.8)	1973–2015	1.5 (0.2 to 2.8)										
15–39	4.1 (2.9 to 5.2)	1973–1991	11.4 (8.8 to 14.1)	1991–2015	−1.1 (−2.0 to −0.3)								
40–64	2.1 (−1.4 to 5.7)	1973–1975	22.4 (−18.9 to 84.5)	1975–1978	−12.2 (−39.2 to 26.8)	1978–1989	8.6 (6.0 to 11.3)	1989–2007	0.6 (−0.2 to 1.3)	2007–2010	−7.7 (−23.6 to 11.5)	2010–2015	2.0 (−2.3 to 6.5)
65–85+	1.7 (0.6 to 2.8)	1973–1978	−2.5 (−8.6 to 3.9)	1978–1991	7.3 (6.0 to 8.6)	1991–1996	−1.5 (−6.3 to 3.5)	1996–2003	4.0 (1.6 to 6.5)	2003–2015	−2.3 (−3.0 to −1.6)		
**Sex**													
Male	1.9 (0.8 to 3.0)	1973–1978	−2.7 (−10.1 to 5.2)	1978–1991	8.2 (6.6 to 9.8)	1991–2005	0.5 (−0.4 to 1.4)	2005–2015	−1.8 (−2.9 to −0.7)				
Female	1.4 (−0.5 to 3.4)	1973–1981	−0.3 (−4.9 to 4.6)	1981–1985	13.5 (−4.5 to 34.8)	1985–2004	2.1 (1.3 to 3.0)	2004–2015	−2.6 (−4.0 to −1.1)				
**Race**													
White	1.7 (0.7 to 2.6)	1973–1980	−0.5 (−4.1 to 3.3)	1980–1990	9.0 (7.0 to 11.1)	1990–1997	−0.7 (−3.3 to 1.9)	1997–2003	3.5 (0.3 to 6.8)	2003–2015	−2.4 (−3.2 to −1.6)		
Black	1.1 (−0.2 to 2.4)	1973–2005	2.9 (1.6 to 4.2)	2005–2015	−4.3 (−8.0 to −0.4)								
Other	2.5 (1.1 to 3.9)	1973–1993	5.7 (2.9 to 8.6)	1993–2015	−0.4 (−1.4 to 0.7)								
**Ann Arbor Stage**													
I/II	0.9 (0.1 to 1.7)	1983–1990	5.7 (2.7 to 8.8)	1990–2003	1.7 (0.8 to 2.7)	2003–2015	−2.8 (−3.7 to −1.9)						
III/IV	2.5 (1.0 to 4.1)	1983–1990	8.7 (2.6 to 15.1)	1990–2007	1.9 (0.8 to 3.1)	2007–2015	−1.3 (−4.2 to 1.6)						
Unknown	−1.8 (−2.9 to −0.6)	1983–2015	−1.8 (−2.9 to −0.6)										
**Site**													
Head/Neck	2.3 (1.2 to 3.4)	1973–1986	7.4 (4.4 to 10.5)	1986–2003	1.7 (0.4 to 3.0)	2003–2015	−2.1 (−3.7 to −0.4)						
Skin and soft tissue	2.3 (1.2 to 3.3)	1973–2000	5.5 (4.1 to 6.8)	2000–2015	−3.2 (−5.1 to −1.4)								
Gastrointestinal tract	0.1 (−1.1 to 1.4)	1973–1978	−4.6 (−12.2 to 3.6)	1978–1987	9.3 (5.8 to 12.9)	1987–2005	−0.3 (−1.0 to 0.5)	2005-2015	−4.5 (−6.1 to −2.8)				
Genitourinary tract	2.3 (1.2 to 3.3)	1973–2003	4.3 (3.2 to 5.5)	2003–2015	−2.7 (−5.2 to −0.1)								
Skeletal tissue	2.0 (−1.4 to 5.5)	1973–1981	−10.2 (−21.7 to 2.9)	1981–1991	13.7 (3.6 to 24.7)	1991–2015	1.7 (0.4 to 3.0)						
Respiratory system	4.2 (0.4 to 8.1)	1973–1985	2.3 (-5.8 to 11.1)	1985–1991	24.1 (1.0 to 52.6)	1991–2015	0.7 (−0.5 to 1.8)						
Hematologic system	8.2 (6.0 to 10.5)	1974–1992	13.3 (8.1 to 18.7)	1992–2015	4.4 (3.1 to 5.7)								
Liver/pancreas	4.8 (3.1 to 6.6)	1974–2001	8.3 (5.8 to 10.9)	2001–2015	−1.6 (−4.1 to 1.0)								
Breast tissue	2.1 (1.2 to 3.1)	1973–2015	2.1 (1.2 to 3.1)										
Other	−0.5 (−7.1 to 6.7)	1973–1982	−18.4 (−29.1 to −6.1)	1982–1986	36.9 (−30.6 to 169.8)	1986–2015	1.3 (−0.0 to 2.7)						

**Table 3:** Trends in extranodal diffuse large B-cell lymphoma incidence-based mortality rates (1988–2015): the SEER-9 registry database.

		**Trend**									
	**Overall (1988–2015)**		**1**			**2**			**3**		
	**APC (95% CI)**	***P*-value**	**Year**	**APC (95% CI)**	***P*-value**	**Year**	**APC (95% CI)**	***P*-value**	**Year**	**APC (95% CI)**	***P*-value**
**Overall**	0.9 (0.1 to 1.8)	0.0	1988–1993	7.2 (2.4 to 12.1)	0.0	1993–2015	−0.4 (−0.8 to −0.0)	0.0			
**Age, y**											
≤14	−1.6 (−2.5 to −0.7)	0.0	1988–2003	−1.6 (−2.5 to −0.7)	0.0						
15–39	−3.6 (−8.2 to 1.2)	0.1	1988–1993	17.0 (3.1 to 32.9)	0.0	1993–1997	−19.5 (−39.5 to 7.1)	0.1	1997–2015	−5.0 (−7.7 to −2.1)	0.0
40–64	−1.6 (−2.8 to −0.4)	0.0	1988–1995	5.7 (1.1 to 10.5)	0.0	1995–2015	−4.0 (−4.8 to −3.2)	0.0			
65–85+	1.2 (0.7 to 1.7)	0.0	1988–2002	2.9 (2.1 to 3.7)	0.0	2002–2015	−0.6 (−1.3 to 0.1)	0.1			
**Sex**											
Male	0.9 (0.0 to 1.8)	0.0	1988–1993	7.1 (2.3 to 12.2)	0.0	1993–2015	−0.5 (−0.9 to −0.1)	0.0			
Female	0.6 (-0.2 to 1.4)	0.1	1988–2001	2.7 (1.3 to 4.1)	0.0	2001–2015	−1.3 (−2.3 to −0.3)	0.0			
**Race**											
White	0.9 (−0.1 to 2.0)	0.1	1988–1991	9.7 (0.7 to 19.5)	0.0	1991–2002	0.9 (−0.1 to 2.0)	0.1	2002–2015	−1.1 (−1.7 to −0.4)	0.0
Black	−0.5 (−1.8 to 0.9)	0.5	1988–2015	−0.5 (−1.8 to 0.9)	0.5						
Other	−0.0 (−0.9 to 0.9)	1.0	1988–2015	−0.0 (−0.9 to 0.9)	1.0						
**Ann Arbor Stage**											
I/II	0.9 (0.1 to 1.7)	0.0	1988–2000	4.0 (2.4 to 5.5)	0.0	2000–2015	−1.5 (−2.3 to −0.6)	0.0			
III/IV	2.4 (−0.2 to 5.2)	0.1	1988–1990	28.5 (−11.3 to 86.0)	0.2	1990–2015	0.6 (0.1 to 1.1)	0.0			
Unknown	−4.2 (−5.1 to −3.3)	0.0	1988–2015	−4.2 (−5.1 to −3.3)	0.0						
**Site**											
Head/Neck	0.7 (−0.9 to 2.3)	0.4	1988–1993	9.2 (0.2 to 19.1)	0.0	1993–2015	−1.2 (−1.9 to −0.4)	0.0			
Skin and soft tissue	1.3 (−0.4 to 2.9)	0.1	1988–2004	3.3 (1.2 to 5.5)	0.0	2004–2015	−1.6 (−4.5 to 1.3)	0.3			
Gastrointestinal tract	−0.6 (−1.6 to 0.4)	0.2	1988–1993	4.6 (−0.7 to 10.2)	0.1	1993–2015	−1.8 (−2.3 to −1.3)	0.0			
Genitourinary tract	1.2 (−0.3 to 2.6)	0.1	1988–2015	1.2 (−0.3 to 2.6)	0.1						
Skeletal tissue	0.8 (−0.5 to 2.2)	0.2	1988–2015	0.8 (−0.5 to 2.2)	0.2						
Respiratory system	1.0 (−0.8 to 2.8)	0.3	1988–2015	1.0 (−0.8 to 2.8)	0.3						
Hematologic system	4.2 (2.6 to 5.7)	0.0	1988–2015	4.2 (2.6 to 5.7)	0.0						
Liver/pancreas	−0.1 (−3.6 to 3.5)	0.9	1988–2011	3.4 (1.4 to 5.5)	0.0	2011–2015	−18.4 (−34.9 to 2.4)	0.1			
Breast tissue	0.3 (−1.4 to 1.9)	0.8	1989–2015	0.3 (−1.4 to 1.9)	0.8						
Other	2.1 (0.6 to 3.6)	0.0	1988–2015	2.1 (0.6 to 3.6)	0.0						

### Overall Incidence and Mortality Trends

**Figure 1:**
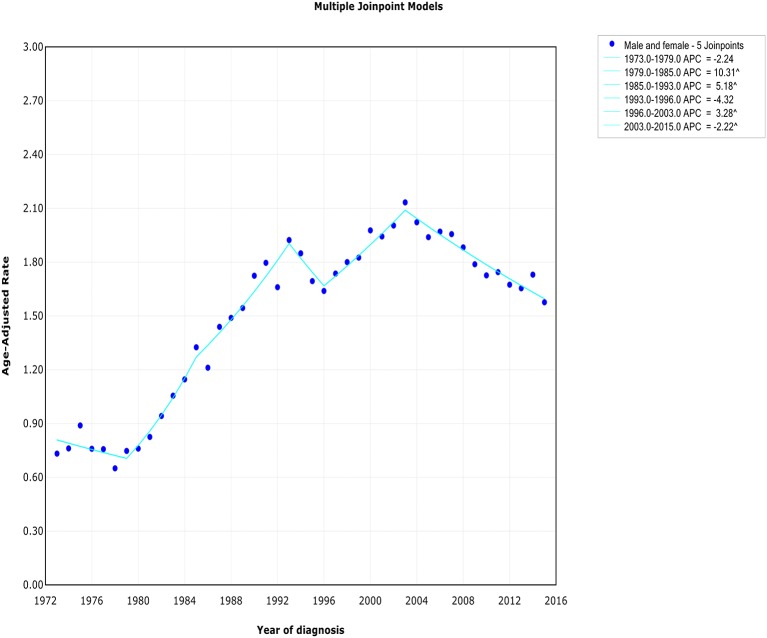
The overall trends of incidence of extranodal DLBCL.

The extranodal DLBCL incidence continued to rise at a rate of 1.6% (95% CI, 0.4–2.8, *p* < 0.001) per year over study period, until it declined around 2003 (Table 2). The incidence of extranodal DLBCL increased steeply during 1996–2003 (APC, 3.3% [95% CI, 1.4–5.2%]), whereas it turned to decrease during 2003–2015 (APC, −2.2% [95% CI, −2.8 to −1.6%]) (Figure 1). The incidence-based mortality trend of extranodal DLBCL had a similar pattern, with a decrease happening around 1993 (Table 3). The annual percentage rates of incidence-based mortality during 1988–1993 was 7.2% (95% CI, 2.4–12.1%), whereas during 1993–2015 the rate was −0.4% (95% CI, −0.8 to −0.0%]) (Figure S1).

### Trends by Sex

**Figure 2:**
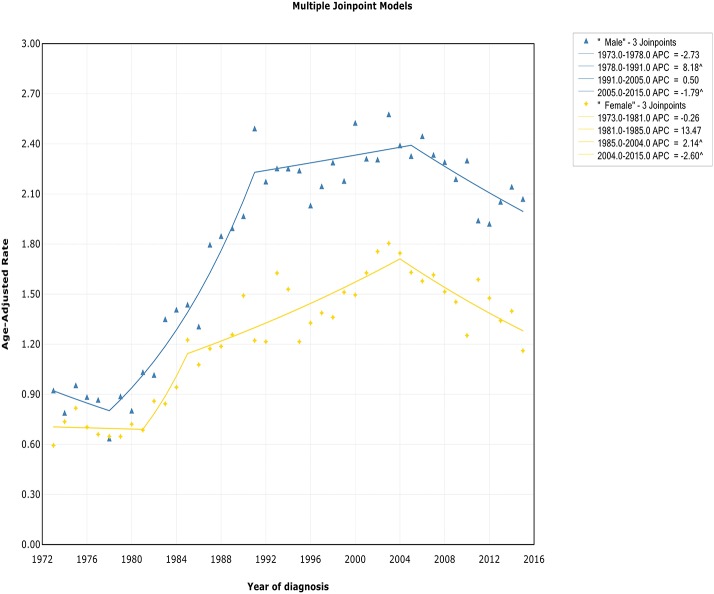
Trends of annual incidence of extranodal DLBCL according to gender.

The incidence rate in males showed a significant increase from 1978 to 1991 (APC, 8.2% [95% CI, 6.6–9.8%]), and then it stabilized from 1991 to 2005 (APC, 0.5% [95% CI, −0.4 to 1.4%]), followed by a sharp decrease from 2005 to 2015 (APC, −1.8% [95% CI, −2.9 to −0.7%]) (Figure 2). The incidence in females had a similar pattern, with a deceleration around 2004 (APC, −2.6% [95% CI, −4.0 to −1.1%]). The incidence-based mortality of both males and females initially increased rapidly, followed by declines at a rate of −0.5% (95% CI, −0.9 to −0.1) and −1.3% (95% CI, −2.3 to −0.3) in 1993 and 2001, respectively (Figure S2). It's worth noting that the annual incidence and mortality rates in men are ~2 times that in women (i.e., 2.49 vs. 1.22 cases per 100,000 in men vs. women in 1991 for incidence; 1.70 vs. 0.87 cases per 100,000 in men vs. women in 1995 for mortality), and the trends have similar slopes. In addition, the incidence and death rates for men are increasing at about 1.5 times the rate for women per year throughout the study period (i.e., 1.9 vs. 1.4%; 0.9 vs. 0.6%).

### Trends by Stage

**Figure 3:**
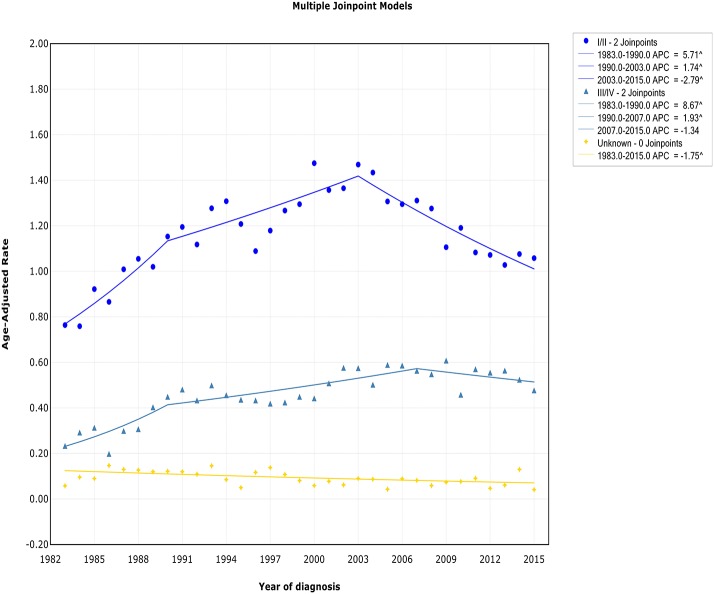
Trends of annual incidence of extranodal DLBCL according to stage.

Both the incidence and incidence-based mortality of early stage patients showed an initial significant increase and then turn to decrease. The incidence increased steeply during 1983–1990 (APC, 5.7% [95% CI, 2.7–8.8%]) and more slowly from 1990 to 2003 (APC, 1.7% [95% CI, 0.8–2.7%]), then it went down during 2003–2015 (APC, −2.8% [95% CI, −3.7 to −1.9%]) (Figure 3). In terms of IB mortality, it initially grew at a rapid rate of 4.0% (95% CI, 2.4–5.5) from 1988 to 2000, followed by declined at a rate of −1.5% (95% CI, −2.3 to −0.6) from 2000 to 2015 (Figure S3). Therefore, the incidence-based mortality rate declined 3 years earlier than the incidence rate

For advanced stage patients, the incidence and incidence-based mortality rates started from a rapid increase, with a change occurring 2007 and 1990, respectively, that the incidence rate turned to decrease and mortality rates leveled off.

### Trends by Age

**Figure 4:**
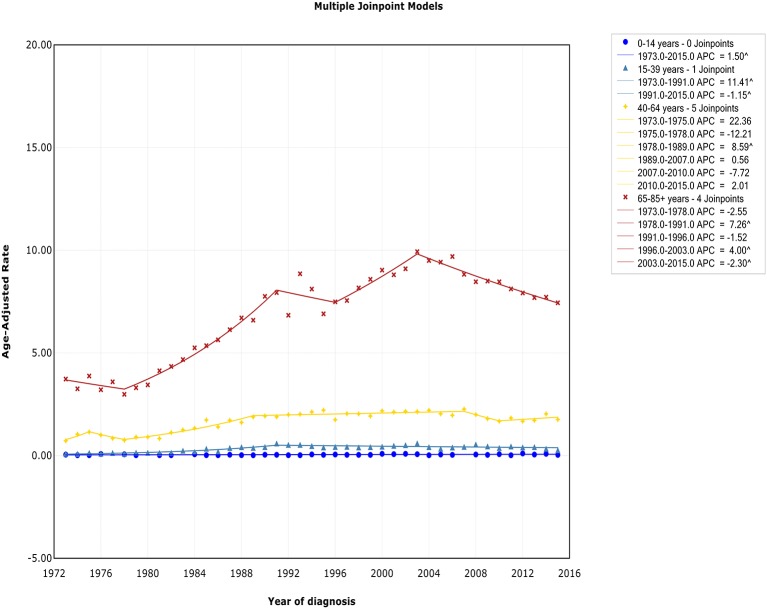
Trends of annual incidence of extranodal DLBCL according to age.

There was no joinpoint in the trend of incidence and incidence-based mortality in the children over the study periods, which rose at a rate of 1.5% (95% CI, 0.2–2.8) and fell at a rate of −1.6% (95% CI, −2.5 to −0.7) per year, respectively. For the AYA and the elderly, the incidence showed an initial increase, followed by declines at a rate of −1.1% (95% CI, −2.0 to −0.3) and −2.3% (95% CI, −3.0 to −1.6) around 1991 and 2003, respectively (Figure 4). The incidence-based mortality for the AYA and the elderly had a similar pattern, with a decrease happening around 1993 and 2002, respectively. For the adults, the incidence has been on the rise over study period, except for a brief decline from 2007 to 2010, whereas the incidence-based mortality has been on a downward trend since 1995 (Figure S4). It was worth noting that the incidence in the elderly was about 4.6 times that of adults, 16.6 times that of AYA, and more than 141.9 times that of children (i.e., 9.93 vs. 2.14 cases per 100,000 in elderly vs. adults in 2003; 9.93 vs. 0.60 cases per 100,000 in elderly vs. AYA in 2003; 9.93 vs. 0.07 cases per 100,000 in elderly vs. children in 2003). The trends have similar slopes. But the increasing rate per year in AYA incidence was the fastest throughout the study period, and was about 2.4 times the rate for elderly (i.e., 4.1 vs. 1.7%).

### Trends by Race

**Figure 5:**
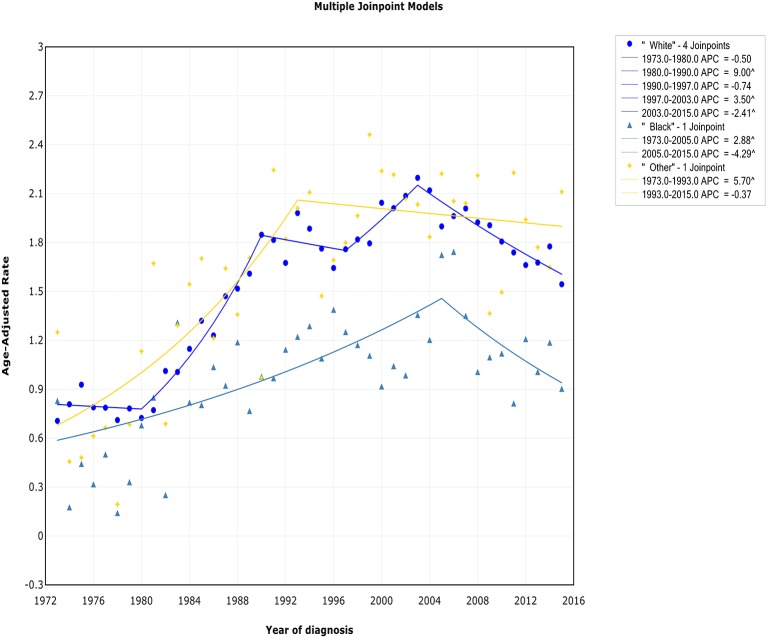
Trends of annual incidence of extranodal DLBCL according to race.

There was a continued increase in both incidence and incidence-based mortality of white patients, with a decrease happening 2003 and 2002, respectively. The incidence of white patients increased steeply during 1980–1990 (APC, 9.0% [95% CI, 7.0–11.1%]) and 1997–2003 (APC, 3.5% [95% CI, 0.3–6.8%]), then it went down during 2003–2015 (APC, −2.4% [95% CI, −3.2 to −1.6%]) (Figure 5). In terms of IB mortality, it increased significantly at a rapid rate of 9.7% (95% CI, 0.7–19.5) during 1988–1991 and turn to level off (APC, 0.9% [95% CI, −0.1 to 2.0%]) from 1991 to 2002, followed by declined at a rate of −1.1% (95% CI, −1.7 to −0.4) from 2002 to 2015 (Figure S5). A turn point was found in incidence for black and other patients around 2005 and 1993, respectively, but no change was found in incidence-based mortality for them.

### Trends by Sites

**Figure 6:**
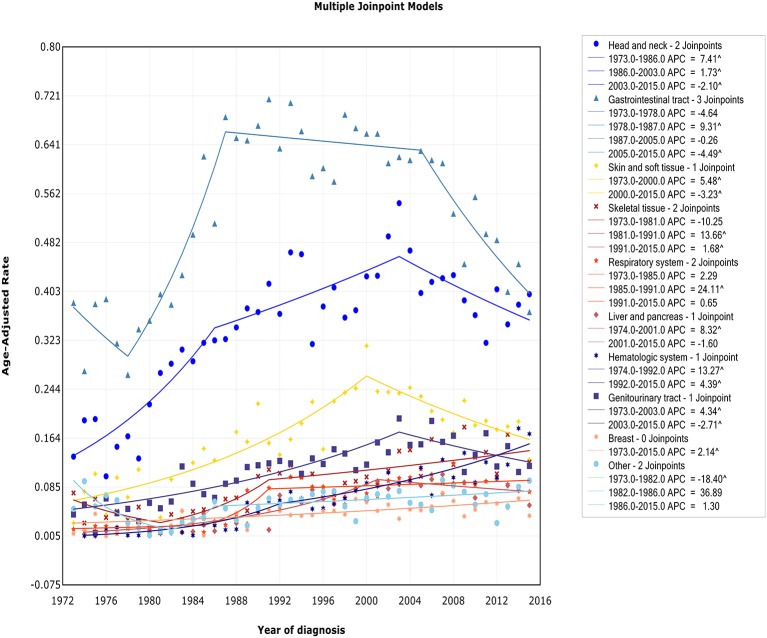
Trends of annual incidence of extranodal DLBCL according to sites.

We observed two different patterns in incidence analysis by sites (Figure 6): (1) After an initial period of substantial and sustained growth, there has been a promising decline in recent years (including sites: head/neck, skin and soft tissue, gastrointestinal tract, genitourinary tract, and liver/pancreas); (2) The incidence almost has been on an upward trend (including sites: skeletal tissue, respiratory system, hematologic system, breast tissue, and other). The incidence-based mortality at each site followed a similar pattern, except for genitourinary tract. There was no turn point in incidence-based mortality in genitourinary tract (Figure S6).

### Survival Analysis

**Figure 7:**
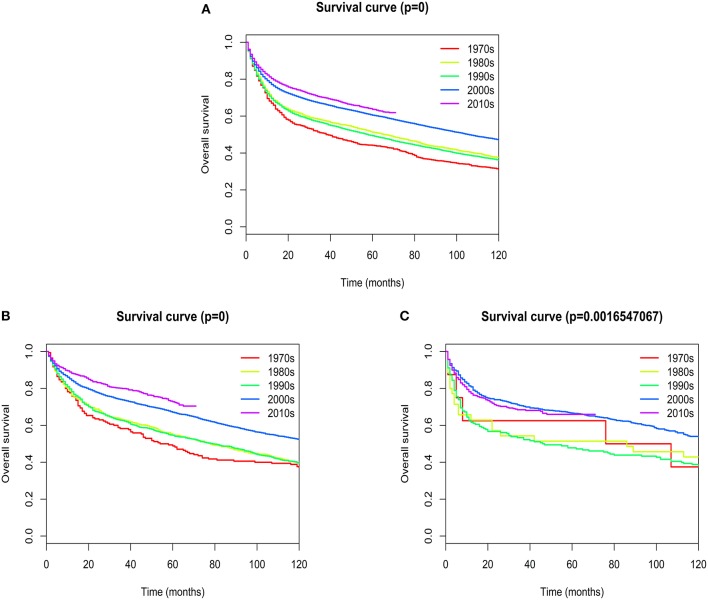
Kaplan–Meier's analysis for extranodal DLBCL. Graph shows increasing survival from the 1970s to 2010s. **(A)** Overall survival analysis; **(B)** Overall survival in Head/Neck; **(C)** Overall survival in Hematologic system.

Overall median survival for patients with extranodal DLBCL was 93 months, with 1-, 5-, and 10- year survival rates of 75.767, 57.937, and 44.021%, respectively. Five-year survival rates improved dramatically from the 1970s to 2010s (44.15 vs. 63.7%; Figure 7A). The most obvious increase of 5-year survival rate occurred in patients with primary site in the head/neck (48.82 vs. 72.76%; Figure 7B). There was a slight, but not statistically significant improvement of 5-year survival rate in patients with primary site in the hematologic system (62.5 vs. 65.95%; Figure 7C). The change of 5-year survival rates from the 1970s to 2010s according to different primary sites were shown graphically in Figure S7.

### Construction and Validation of the Nomograms

A total of 17,744 patients with complete information from the SEER-18 database were included in the construction of the nomograms from 2002 to 2015 (Table S1). Among them, most patients were man, early stage, and white. Gastrointestinal tract, head/neck and skin and soft tissue were the most common primary sites.

On multivariate analysis, male sex, older age, black race, advanced Ann Arbor Stage (III/IV), and primary sites in the skin and soft tissue, gastrointestinal tract, genitourinary tract, respiratory system, liver/pancreas, breast tissue, and other were associated with decreased survival (Table S2).

**Figure 8:**
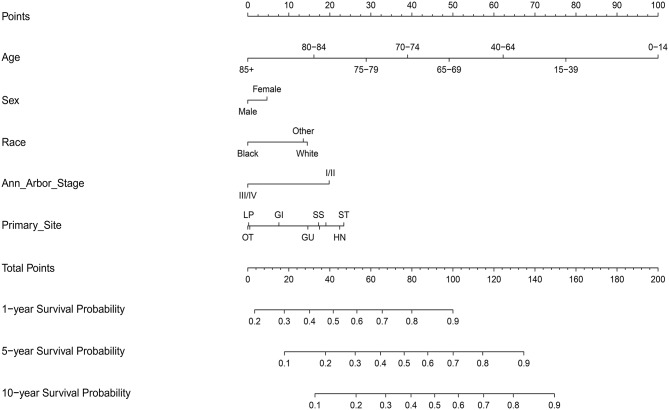
Nomograms of patients with extranodal DLBCL for predicting overall survival.

**Figure 9:**
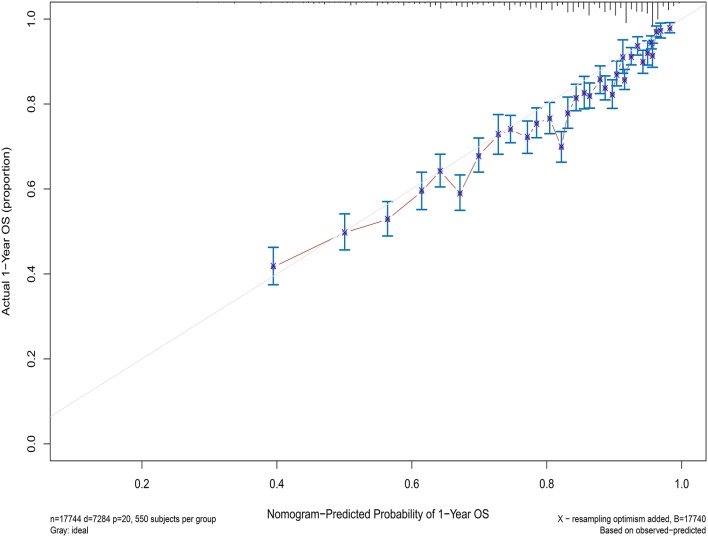
The calibration curves for predictions of overall survival at 1 year after diagnosis.

**Figure 10:**
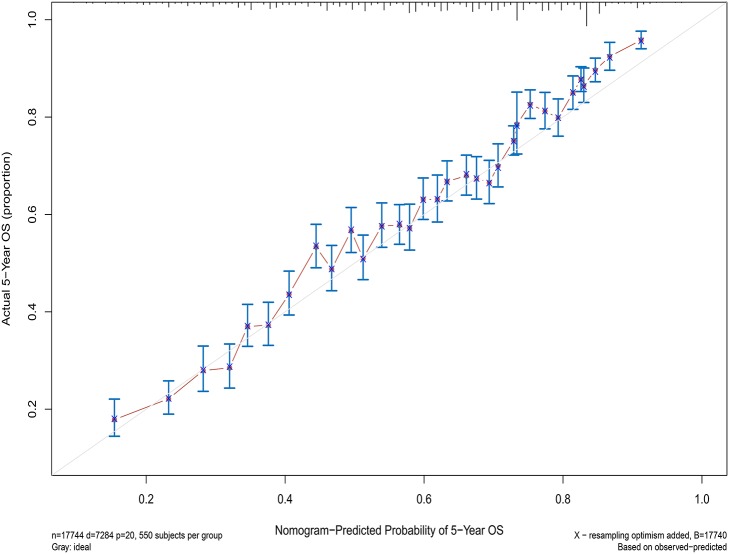
The calibration curves for predictions of overall survival at 5 years after diagnosis.

**Figure 11:**
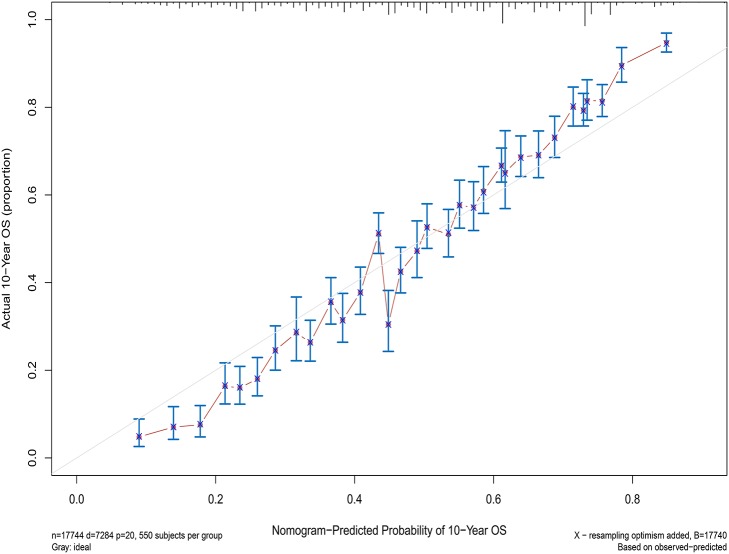
The calibration curves for predictions of overall survival at 10 years after diagnosis.

Next, the factors closely related to survival on multivariate analysis were used to construct nomograms by the R Bioconductor (Figure 8). The prognostic signature for 1-, 5-, and 10-year overall survival is demonstrated in Figure 8. The C-index and the calibration plots were powerful in assessing the performance of a model. To confirm whether the prognostic signature could predict the prognosis of patients with extranodal DLBCL, these two methods were applied. The C-index showed a value for OS of 0.708, which also validated the signature performed well and was able to forecast the prognosis of patients with extranodal DLBCL successfully. The calibration curves showed satisfactory consistency between predicted values of the model and true values for 1-, 5-, and 10-year OS (Figures 9–11).

## Discussion

As far as we know, this is the first ever study based on large population to uncover trend of the incidence and mortality for extranodal DLBCL by clinical features, and to systematically compare them based on these characteristics, which may help scientists identify disease-related risk factors and better manage the disease. The appropriate management of extranodal DLBCL should be able to stratify patients into distinct prognostic groups. Therefore, we have developed a new prediction model in the rituximab era that may provide an accurate risk stratification of individual to determine the best treatment options for the individual. We demonstrate that the incidence and incidence-based mortality of extranodal DLBCL have been increasing for decades, but it has shown a promising downward trend in recent years. This phenomenon may be partly explained by our survival analysis. The 5-year survival rates have improved dramatically from the 1970s to 2010s (44.15 vs. 63.7%), and the most obvious increase occurred in patients with primary site in the head/neck.

The overall mortality trend of extranodal DLBCL began to decline in 1993, which was 10 years earlier than incidence, indicating that the main reason for the decline of extranodal DLBCL mortality is the improvement of survival rate. We hypothesize that improvements in management and treatment of patients may lead to improved survival. One of the most significant improvements was the introduction of the IPI index as the gold standard for classifying high-risk and low-risk patients and guiding treatment (11). Another significant cause was the introduction of rituximab since 1988, which had the potential to cure patients (12). In addition, a deeper and more comprehensive understanding of the genetics and molecular biology of DLBCL, such as BCL2 protein expression (13) and genetic complexity (14), may also be helpful in patient management and aid in improving survival.

It is worth noting that the mortality trend of early stage patients has been declining since 2007, and the mortality rate of advanced stage patients has only stabilized from the initial rapid growth since 1993, but it has not shown a downward trend. This phenomenon may suggest that new, more effective and systemic treatments, in addition to R-CHOP schemes, are needed to prevent the fatal nature of patients at advanced stage. Chimeric antigen receptor modified T (CAR-T) cell therapy by targeting the CD19 antigen has made breakthroughs in the treatment of NHL at advanced stage, and showed the possibility of cure (15). Axicabtagene ciloleucel (16) and tisagenlecleucel (17) have been approved by the FDA. The continued monitoring of extranodal DLBCL mortality may help to assess the effectiveness of clinical approaches and aid the development of new therapeutic approaches.

We here showed the decline of primary gastrointestinal DLBCL mortality since 1993. A large number of studies have shown that *Helicobacter pylori* (*H. pylori*) and *Campylobacter jejuni* was closely related to primary gastrointestinal DLBCL (18–21). In 1993, Wotherspoon found that there was a high incidence of *H. pylori* infection among patients with gastric lymphoma, and 5 out of 6 patients achieved complete remission after eradication of *H. pylori* infection (18, 22). T Chronic enteritis and gastritis secondary to *Campylobacter jejuni* and *H. pylori* has been identified as an important predisposition to primary gastrointestinal lymphoma (23). In recent years, endoscopic ultrasonography could be used to track such patients for a long time, classify the disease stage and eradicate the infection in time (24). These advances may be associated with the large decline in the incidence of primary gastrointestinal DLBCL from 2005. In addition, the dramatic decline in the incidence and mortality of primary liver/pancreas DLBCL patients since 2001 and 2011, respectively, may be related to the following aspects: (1) the recognition that the hepatitis C Virus is a key factor in the development of primary liver DLBCL; (2) the availability of advanced methods for the effective prevention and control of the hepatitis C virus (25–27).

In our analysis, the incidence and mortality of male extranodal DLBCL was twice that of women, and its annual growth rate was about 1.5 times that of women, which was consistent with previous studies (28). This was reminiscent of the fact that for most cancers, men were at high risk. Although the underlying causes were unknown (29), there were several possible reasons for this: (1) After the reporting of the initial phase 3 trials, data suggested that female responded better to rituximab than male (30–32). The speed at which rituximab is cleared in the body is the key cause of this phenomenon (33). The data from RICOVER-60 trial showed that elderly females who gained the best beneficial effects from rituximab had a statistically significant slower clearance of rituximab, which brought about longer exposure time and higher serum levels in relation to an age-dependent decrease in clearance of rituximab in females (32). But elder males have a faster clearance of rituximab which leads to suboptimal dosing with rituximab when dosed at 375 mg/m^2^. (2) The differences were in occupational exposure factors, health awareness, and lifestyle (28). For example, smoking has been shown to be a predisposing factor for non-hodgkin's lymphoma in a dose-dependent manner (34). (3) The immune response has been shown to be closely related to lymphomagenesis (35), and women had stronger humoral and cellular immunity than men (36). (4) Hormonal differences were between men and women. For example, reproductive hormone could regulate the immune response in a variety of ways (37). (5) Men were more susceptible to *H. pylori* infection, which was closely related to primary gastrointestinal lymphoma (38).

In our article, we showed that the incidence of extranodal DLBCL increased with age, which was consistent with the theory that the incidence increased exponentially with age in the multi-step carcinogenic model of solid tumors (39). Two factors may account for this result: (1) With age, the immune system of the elderly gradually declined and the incidence of chronic inflammation gradually increased. For example, the prevalence of EBV virus increased with age. Continuous stimulation of the EBV virus leads to T-cell exhaustion that favors telomere attrition and immune senescence (40). In addition, EBV virus was an important susceptibility factor leading to extranodal DLBCL (41, 42). (2) Some genes and molecules would change with age (14). For example, the elderly were more likely to have higher levels of BCL2 than younger patients (43).

The death rate for white patients has declined significantly since 2002, while that for black and other ethnic groups has remained virtually unchanged. One possible explanation for this was reported by an article showing that the black and other race people had less access to rituximab than white people in 2002, when rituximab was first used (44). Another reason may be that the number of black people and other ethnic groups in our study was too small, hindering the discovery of a significant decline in mortality. More large population-based researches were needed to explore genetic and molecular differences between extranodal DLBCL patients with different races.

We here showed that extranodal DLBCL patients with primary site in the head/neck had the most obvious increase of 5-year survival rate from the 1970s to 2010s (48.82 vs. 72.76%), which is consistent with previous studies (45–47). The possible reasons were listed as following: (1) the knowledge on extranodal lymphoma of the head and neck remained rare from 1970s to 80s (48), while many studies on such subgroup of lymphoma are being published at present considering its heterogeneous nature (47). (2) Rituximab was replenished to conventional CHOP or CHOP-like schemes for DLBCL in the late 1990s, which has led to considerable improvement in patients' survival. Furthermore, the development of novel drugs for lymphoma of distinct pathological types has resulted in a higher probability of cure (49). (3) The head and neck area which could be easily approached and assessed by palpation or endoscopy is a frequent site for tissue confirmation. Surgeons on head and neck are often consulted for biopsies and prognosis judgements of suspicious extranodal lymphomas. These may lead to the phenomenon that primary DLBCL in head and neck could be diagnosed and treated early in the clinical setting. It has been previously reported that DLBCL patients with extranodal sites in the head and neck experienced longer survival than those with nodal lymphoma. Additionally, the extranodal group also had longer disease-specific survival than the nodal group with extranodal involvement of other sites (45).

At present, the most routine prognostic system in DLBCL patients is the International Prognostic Index (IPI). However, there is multiple variables and prognosis heterogeneity in some defined risk groups according to the IPI guidelines. As a graphic expression of a mathematical model, the nomogram helps to determine the possibility of clinical event by combing biological and clinical variables. Nomograms are widely used in various types of cancers (50, 51). There is increasing evidence that primary extranodal sites reflect distinct clinical features and prognostic implications, and require specific therapy (8, 9). The number of extranodal sites is one of the evaluation standard of the IPI score. However, Lu CS et al. had demonstrated that some specific extranodal sites have a better predictive value than the number of extranodal sites involved (7). Therefore, primary site, as an independent prognostic factor by multivariate COX, was added to this model. The prognostic value of this model was verified by C-index and the calibration curves, which are powerful in assessing the performance of a model. The high value (0.708) of C-index for OS indicated that the model may provide the best prognostic function. The Calibration curves displayed a wonderful agreement between the predicted values and the true outcomes, which may ensure the reliability of the risk stratification signature. Compared with IPI, the new risk stratification signature could divide extranodal DLBCL patients into high risk and low risk groups more accurately, which could help decrease over-therapy in low-risk patients and promote effective therapies in high-risk patients to prevent the fatal nature of this disease. Moreover, the nomograms could compute one individual's 1-, 5-, and 10-year survival rates, respectively, and therefore may develop a more rational follow-up schedule with the patient's doctor.

Some limitations due to information availability in the SEER database should be noticed when interpreting our findings. First, information regarding therapy is limited. In SEER, there is no information about chemotherapy, which is a major modality of treatment for DLBCL. Therefore, we couldn't explore the effect of treatment on survival and the trend of incidence and mortality. We partially addressed this issue by dividing the time of diagnosis into 1970s, 1980s, 1990s, 2000s, and 2010s. Second, the incidence-based mortality rates by Ann Arbor Stage may be underestimated, because the SEER database has recorded the information of Ann Arbor Stage since 1983, which shortened the incubation period between diagnosis and death. Third, the information about radiation exposure, environmental exposures and individual life style and family history, which may be associated with the rates of incidence and mortality, was not available. Fourth, there was no record of baseline performance status, B-symptoms, bulky disease, and lactate dehydrogenase levels. Therefore, we couldn't include these potential prognostic factors in the prediction model.

## Conclusion

Our study shows that the incidence and incidence-based mortality of extranodal DLBCL had been increasing for decades, but have shown a promising downward trend in recent years. The findings may provide new insight into better healthcare quality and better manage extranodal DLBCL. Moreover, the 5-year survival rates have improved dramatically from the 1970s to 2010s (44.15 vs. 63.7%), and the most obvious increase occurred in patients with primary site in the head/neck. We also constructed the nomogram, a robust, and clinically practical risk stratification, in the rituximab era, which may help guide treatment and develop individual-specific tracking programs.

## Data Availability Statement

Publicly available datasets were analyzed in this study. This data can be found here: the Surveillance, Epidemiology and End Results database.

## Ethics Statement

This study was conducted in full compliance with the publication guidelines provided by SEER. The data were obtained from SEER, so the approval of an ethics committee was not needed.

## Author Contributions

XY collected and analyzed the data and wrote the paper. AX, FF, ZH, QC, and LZ research literature, edit the paper, and revise the paper. CS and YH conceived and designed this study, analyzed the data, and wrote the paper. All authors reviewed the paper, and approved the final manuscript.

### Conflict of Interest

The authors declare that the research was conducted in the absence of any commercial or financial relationships that could be construed as a potential conflict of interest.

## Supplementary Material

Table S1 Demographics and clinical characteristics of the patients included in the construction of the nomograms (2002–2015): The SEER-18 Registry Database.

Table S2 Univariate and multivariate analysis of prognostic factors for overall survival in extranodal diffuse large B-Cell Lymphoma Patients included in the construction of the nomograms (2002–2015): The SEER-18 Registry Database.

Figure S1 The overall trends of incidence-based mortality of extranodal DLBCL.

Figure S2 Trends of annual incidence-based mortality of extranodal DLBCL according to gender.

Figure S3 Trends of annual incidence-based mortality of extranodal DLBCL according to stage.

Figure S4 Trends of annual incidence-based mortality of extranodal DLBCL according to age.

Figure S5 Trends of annual incidence-based mortality of extranodal DLBCL according to race.

Figure S6 Trends of annual incidence-based mortality of extranodal DLBCL according to sites.

Figure S7 Kaplan–Meier's analysis according to sites. Graph shows increasing survival from the 1970s to 2000s. **(A)** Overall survival in Skin and soft tissue; **(B)** Overall survival in Gastrointestinal tract; **(C)** Overall survival in Skeletal tissue. **(D)** Overall survival in Respiratory system; **(E)** Overall survival in Liver/pancreas; **(F)** Overall survival in Breast tissue; **(G)** Overall survival in Genitourinary tract; **(H)** Overall survival in Other.

## Abbreviations

DLBCL: diffuse large B-cell lymphoma
SEER: Surveillance, Epidemiology, and End Results database
HR: hazard ratio
OS: Overall Survival
R-CHOP: rituximab with cyclophosphamide, doxorubicin, vincristine, and prednisone
C-index: The concordance index
IPI: the International Prognostic Index
*H. pylori*: *Helicobacter pylori*
IBM: incidence-based mortality.
