# Supplementary material for: Incidence and Mortality Trends and Risk Prediction Nomogram for Extranodal Diffuse Large B-Cell Lymphoma: An Analysis of the Surveillance, Epidemiology, and End Results Database
Source: Front Oncol. 2019 Nov 12;9:1198. doi: 10.3389/fonc.2019.01198 (PMC6861389; doi:10.3389/fonc.2019.01198)
Supplement: Table S1 — Demographics and clinical characteristics of the patients included in the construction of the nomograms (2002–2015): The SEER-18 Registry Database. [file Table_1.DOCX]

TABLE S1. Demographics and clinical characteristics of the patients included in the construction of the nomograms(2002-2015): The SEER-18 Registry Database

| **Characteristic** | **Cases,**  **No.** | **%** |
| --- | --- | --- |
| **Overall** | 17744 |  |
| **Age,y** |  |  |
| ≤ 14 | 124 | 0.70 |
| 15-39 | 1432 | 8.07 |
| 40-64 | 6726 | 37.91 |
| 65-69 | 1974 | 11.12 |
| 70-74 | 2130 | 12.00 |
| 75-79 | 2070 | 11.67 |
| 80-84 | 1756 | 9.90 |
| 85+ | 1532 | 8.63 |
| **Sex** |  |  |
| Male | 9813 | 55.3 |
| Female | 7931 | 44.7 |
| **Race** |  |  |
| White | 14715 | 82.93 |
| Black | 1248 | 7.03 |
| Other | 1781 | 10.04 |
| **Ann Arbor stage** |  |  |
| I/II | 12372 | 69.72 |
| III/IV | 5372 | 30.28 |
| **Site** |  |  |
| Head/Neck | 4215 | 23.75 |
| Skin and soft tissue | 1775 | 10.00 |
| Gastrointestinal tract | 5260 | 29.64 |
| Genitourinary tract | 1457 | 8.21 |
| Skeletal tissue | 1360 | 7.66 |
| Respiratory system | 822 | 4.63 |
| Hematologic system | 1126 | 6.35 |
| Liver/pancreas | 766 | 4.32 |
| Breast tissue | 513 | 2.89 |
| Other | 450 | 2.54 |
