# Supplementary material for: Incidence and Mortality Trends and Risk Prediction Nomogram for Extranodal Diffuse Large B-Cell Lymphoma: An Analysis of the Surveillance, Epidemiology, and End Results Database
Source: Front Oncol. 2019 Nov 12;9:1198. doi: 10.3389/fonc.2019.01198 (PMC6861389; doi:10.3389/fonc.2019.01198)
Supplement: Table S2 — Univariate and multivariate analysis of prognostic factors for overall survival in extranodal diffuse large B-Cell Lymphoma Patients included in the construction of the nomograms (2002–2015): The SEER-18 Registry Database. [file Table_2.DOCX]

TABLE S2. Univariate and Multivariate Analysis of Prognostic Factors for Overall Survival in Extranodal Diffuse Large B-Cell Lymphoma Patients included in the construction of the nomograms(2002-2015): The SEER-18 Registry Database

| **Variable** | **Univariate analysis** |  | **Multivariate analysis** |  |
| --- | --- | --- | --- | --- |
|  | **HR (95% CI)** | **P** | **HR (95% CI)** | **P** |
| **Age,y** |  |  |  |  |
| ≤ 14 | 1 | Ref. | 1 | Ref. |
| 15-39 | 2.323(1.193-4.522) | 0.000 | 2.319(1.191-4.514) | 0.013 |
| 40-64 | 4.076(2.117-7.847) | 0.000 | 4.019(2.087-7.739) | 0.000 |
| 65-69 | 6.256(3.242-12.073) | 0.000 | 6.222(3.222-12.013) | 0.000 |
| 70-74 | 8.453(4.385-16.296) | 0.000 | 8.594(4.456-16.576) | 0.000 |
| 75-79 | 11.269(5.848-21.712) | 0.000 | 11.488(5.959-22.149) | 0.000 |
| 80-84 | 15.796(8.198-30.437) | 0.000 | 16.300(8.454-31.427) | 0.000 |
| 85+ | 23.863(12.384-45.983) | 0.000 | 25.734(13.345-49.625) | 0.000 |
| **Sex** |  |  |  |  |
| Male | 1 | Ref. | 1 | Ref. |
| Female | 1.023(0.977--1.071) | 0.332 | 0.866(0.826-0.909) | 0.000 |
| **Race** |  |  |  |  |
| White | 1 | Ref. | 1 | Ref. |
| Black | 0.977(0.892-1.070) | 0.617 | 1.481(1.350-1.625) | 0.000 |
| Other | 0.965(0.891-1.044) | 0.371 | 1.021(0.943-1.105) | 0.616 |
| **Ann Arbor stage** |  |  |  |  |
| I/II | 1 | Ref. | 1 | Ref. |
| III/IV | 1.652(1.575-1.732) | 0.000 | 1.627(1.549-1.708) | 0.000 |
| **Site** |  |  |  |  |
| Head/Neck | 1 | Ref. | 1 | Ref. |
| Skin and soft tissue | 1.343(1.231-1.465) | 0.000 | 1.164 (1.066-1.270) | 0.000 |
| Gastrointestinal tract | 1.508(1.413-1.609) | 0.000 | 1.364(1.277-1.457) | 0.001 |
| Genitourinary tract | 1.319(1.200-1.449) | 0.000 | 1.235(1.123-1.358) | 0.000 |
| Skeletal tissue | 0.801(0.715-0.897) | 0.000 | 0.936(0.835-1.049) | 0.256 |
| Respiratory system | 1.811(1.624-2.019) | 0.000 | 1.683(1.507-1.879) | 0.000 |
| Hematologic system | 1.195(1.070-1.333) | 0.002 | 1.037(0.927-1.160) | 0.521 |
| Liver/pancreas | 1.827(1.632-2.046) | 0.000 | 1.621(1.446-1.816) | 0.000 |
| Breast tissue | 1.207(1.042-1.399) | 0.012 | 1.168(1.007-1.356) | 0.041 |
| Other | 1.927(1.680-2.210) | 0.000 | 1.718(1.497-1.972) | 0.000 |

HR, hazard ratio; CI, confidence interval
