# Supplementary figures and images for: Incidence and Mortality Trends and Risk Prediction Nomogram for Extranodal Diffuse Large B-Cell Lymphoma: An Analysis of the Surveillance, Epidemiology, and End Results Database
Source: Front Oncol. 2019 Nov 12;9:1198. doi: 10.3389/fonc.2019.01198 (PMC6861389; doi:10.3389/fonc.2019.01198)

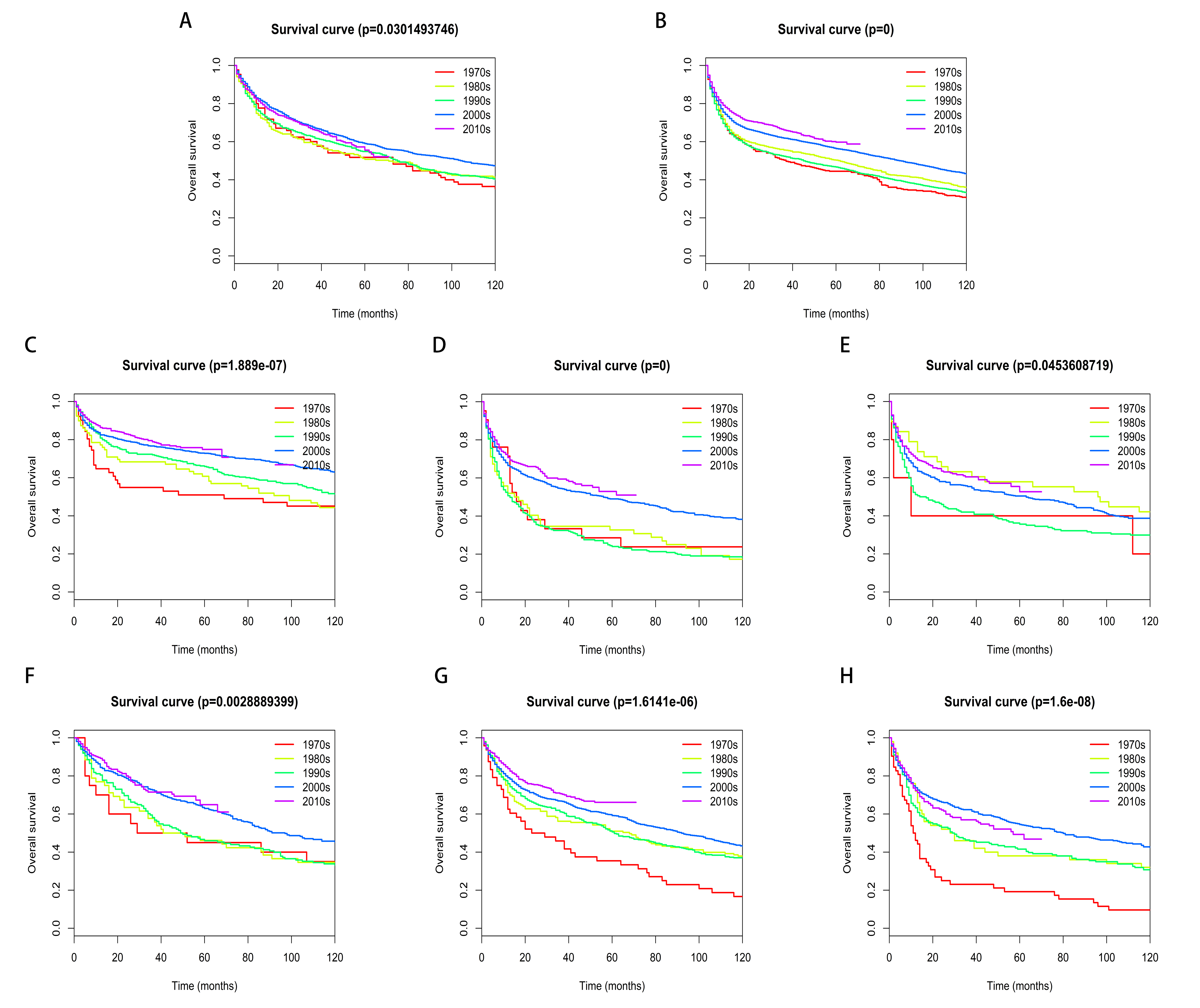

Supplement: Figure S7 — Kaplan–Meier's analysis according to sites. Graph shows increasing survival from the 1970s to 2000s. (A) Overall survival in Skin and soft tissue; (B) Overall survival in Gastrointestinal tract; (C) Overall survival in Skeletal tissue. (D) Overall survival in Respiratory system; (E) Overall survival in Liver/pancreas; (F) Overall survival in Breast tissue; (G) Overall survival in Genitourinary tract; (H) Overall survival in Other. [file Image_7.TIF]
